# The first set of EST resource for gene discovery and marker development in pigeonpea (*Cajanus cajan *L.)

**DOI:** 10.1186/1471-2229-10-45

**Published:** 2010-03-11

**Authors:** Nikku L Raju, Belaghihalli N Gnanesh, Pazhamala Lekha, Balaji Jayashree, Suresh Pande, Pavana J Hiremath, Munishamappa Byregowda, Nagendra K Singh, Rajeev K Varshney

**Affiliations:** 1 International Crops Research Institute for the Semi-Arid Tropics (ICRISAT), Patancheru, Greater Hyderabad 502 324, Andhra Pradesh, India; 2 University of Agricultural Sciences, Gandhi Krishi Vignyan Kendra (GKVK), Bangalore, 560 065, Karnataka, India; 3 National Research Centre on Plant Biotechnology (NRCPB), Indian Agricultural Research Institute, New Delhi 110 012, India; 4 Genomics towards Gene Discovery Sub Programme, Generation Challenge Programme (GCP) c/o CIMMYT, Int. Apartado Postal 6-641, 06600, Mexico, DF Mexico

## Abstract

**Background:**

Pigeonpea (*Cajanus cajan *(L.) Millsp) is one of the major grain legume crops of the tropics and subtropics, but biotic stresses [*Fusarium *wilt (FW), sterility mosaic disease (SMD), etc.] are serious challenges for sustainable crop production. Modern genomic tools such as molecular markers and candidate genes associated with resistance to these stresses offer the possibility of facilitating pigeonpea breeding for improving biotic stress resistance. Availability of limited genomic resources, however, is a serious bottleneck to undertake molecular breeding in pigeonpea to develop superior genotypes with enhanced resistance to above mentioned biotic stresses. With an objective of enhancing genomic resources in pigeonpea, this study reports generation and analysis of comprehensive resource of FW- and SMD- responsive expressed sequence tags (ESTs).

**Results:**

A total of 16 cDNA libraries were constructed from four pigeonpea genotypes that are resistant and susceptible to FW ('ICPL 20102' and 'ICP 2376') and SMD ('ICP 7035' and 'TTB 7') and a total of 9,888 (9,468 high quality) ESTs were generated and deposited in dbEST of GenBank under accession numbers GR463974 to GR473857 and GR958228 to GR958231. Clustering and assembly analyses of these ESTs resulted into 4,557 unique sequences (unigenes) including 697 contigs and 3,860 singletons. BLASTN analysis of 4,557 unigenes showed a significant identity with ESTs of different legumes (23.2-60.3%), rice (28.3%), *Arabidopsis *(33.7%) and poplar (35.4%). As expected, pigeonpea ESTs are more closely related to soybean (60.3%) and cowpea ESTs (43.6%) than other plant ESTs. Similarly, BLASTX similarity results showed that only 1,603 (35.1%) out of 4,557 total unigenes correspond to known proteins in the UniProt database (≤ 1E-08). Functional categorization of the annotated unigenes sequences showed that 153 (3.3%) genes were assigned to cellular component category, 132 (2.8%) to biological process, and 132 (2.8%) in molecular function. Further, 19 genes were identified differentially expressed between FW- responsive genotypes and 20 between SMD- responsive genotypes. Generated ESTs were compiled together with 908 ESTs available in public domain, at the time of analysis, and a set of 5,085 unigenes were defined that were used for identification of molecular markers in pigeonpea. For instance, 3,583 simple sequence repeat (SSR) motifs were identified in 1,365 unigenes and 383 primer pairs were designed. Assessment of a set of 84 primer pairs on 40 elite pigeonpea lines showed polymorphism with 15 (28.8%) markers with an average of four alleles per marker and an average polymorphic information content (PIC) value of 0.40. Similarly, *in silico *mining of 133 contigs with ≥ 5 sequences detected 102 single nucleotide polymorphisms (SNPs) in 37 contigs. As an example, a set of 10 contigs were used for confirming *in silico *predicted SNPs in a set of four genotypes using wet lab experiments. Occurrence of SNPs were confirmed for all the 6 contigs for which scorable and sequenceable amplicons were generated. PCR amplicons were not obtained in case of 4 contigs. Recognition sites for restriction enzymes were identified for 102 SNPs in 37 contigs that indicates possibility of assaying SNPs in 37 genes using cleaved amplified polymorphic sequences (CAPS) assay.

**Conclusion:**

The pigeonpea EST dataset generated here provides a transcriptomic resource for gene discovery and development of functional markers associated with biotic stress resistance. Sequence analyses of this dataset have showed conservation of a considerable number of pigeonpea transcripts across legume and model plant species analysed as well as some putative pigeonpea specific genes. Validation of identified biotic stress responsive genes should provide candidate genes for allele mining as well as candidate markers for molecular breeding.

## Background

Pigeonpea (*Cajanus cajan *(L.) Millsp) is one of the major grain legume crops of the tropical and subtropical regions of the world [1]. It is the only cultivated food crop of the *Cajaninae *sub-tribe and has a diploid genome with 11 pairs of chromosomes (2n = 2× = 22) and a genome size estimated to be 858 Mbp [2]. The genus *Cajanus *comprises 32 species most of which are found in India, Australia and one is native to West Africa. Pigeonpea is a major food legume crop in South Asia and East Africa with India as the largest producer (3.5 Mha) followed by Myanmar (0.54 Mha) and Kenya (0.20 Mha) [3]. It plays an important role in food security, balanced diet and alleviation of poverty because of its diverse usages as a food; fodder and fuel wood [4]. Several abiotic (e.g. drought, salinity and water-logging) and biotic (e.g. diseases like *Fusarium *wilt, sterility mosaic and pod borer insects) stresses, are serious challenges for sustainable pigeonpea production to meet the demands of the resource poor people of several African and Asian countries.

*Fusarium *wilt (FW) caused by *Fusarium udum *is an important biotic constraint in pigeonpea production in the Indian subcontinent, which results in 16-47% crop losses [5]. The fungus enters the host vascular system at the root tips through wounds or invasion made by nematodes, leading to progressive chlorosis of leaves, branches, wilting and collapse of the root system [6]. In India alone, the loss due to this disease is estimated to be US $71 million and the percentage of disease incidence varies from 5.3 to 22.6% [7].

Sterility mosaic disease (SMD) caused by pigeonpea sterility mosaic virus (PPSMV) is one of the wide-spread diseases of pigeonpea, which is transmitted by an eriophyid mite (*Aceria cajani *Channabasavanna). The disease is characterized by the symptoms like bushy and pale green appearance of plants followed by reduction in size, increase in number of secondary and mosaic mottling of leaves and finally partial or complete cessation of reproductive structures. Some parts of the plant may show disease symptoms and other parts may remain unaffected [8].

Due to the above mentioned factors combined with limited water resources to the fields in the semi-arid tropic regions, where the crop is grown, the productivity has remained stagnant at around 0.7 t/ha during the past two decades [1]. With the advent of genomic tools such as molecular markers, genetic maps, etc., conventional plant breeding has been facilitated greatly and improved genotypes/varieties with enhanced resistance/tolerance to biotic/abiotic stresses have been developed in several crop species [9,10]. In case of pigeonpea, however, a very limited number of genomic tools are available so far [11,12]. For instance, 156 microsatellite or simple sequence repeat (SSR) markers [13-16], 908 expressed sequence tags (ESTs), at the time of undertaking the study, were available in pigeonpea. For enhancing the genomic resources in pigeonpea, transcriptome sequencing to generate ESTs should be a fast approach. ESTs, which are generated by large-scale single pass sequencing of randomly picked cDNA clones, have been cost - effective and valuable resource for efficient and rapid identification of novel genes and development of molecular markers [17]. Further, ESTs have been employed in bioinformatic analyses to identify the genes that are differentially expressed in various tissues, cell types, or developmental stages of the same or different genotypes [18,19].

In view of above facts, this study was undertaken to obtain a comprehensive resource of FW- and SMD-responsive ESTs in pigeonpea with the following objectives: (i) generation of FW- and SMD- responsive ESTs, (ii) functional annotation of assembled unigenes, (iii) *in silico *identification of putative FW- and SMD- responsive genes, and (iv) development of novel SSR and SNP markers in pigeonpea.

## Results

**Figure 1:**
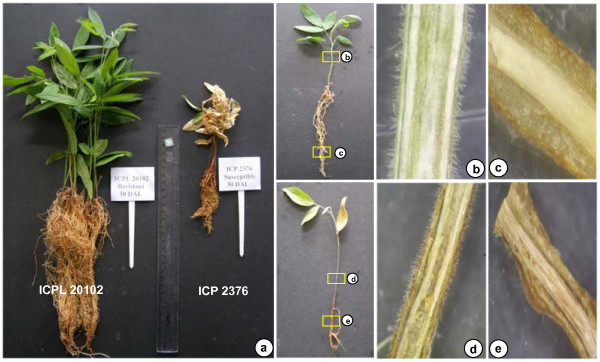
***Fusarium *wilt (FW) challenged pigeonpea seedlings at 30 days after inoculation (DAI)**. a) *Fusarium *wilt challenged pigeonpea genotypes ('ICPL 20102') and ('ICP 2376') at 30 days after inoculation (30 DAI); b & c) Microscopic examination of FW-resistant pigeonpea genotype ('ICPL 20102') showing no disease symptoms on shoot and root vascular tissues; d & e) Microscopic examination of FW-susceptible pigeonpea genotype ('ICP 2376') showing severe wilt symptoms on shoot and root vascular tissues.

Root tissue is the site for *Fusarium udum *infection, the causal fungal agent of *Fusarium *wilt in pigeonpea. With an objective to evaluate the transcriptional responses after infection of roots by *F. udum*, six unidirectional cDNA libraries were constructed. These are from each of FW- infected root tissues of resistant ('ICPL 20102') and susceptible ('ICP 2376') genotypes at different stages *viz*. 6, 10, 15, 20, 25, 30 days after inoculation (DAI). Infected roots were examined by light microscopy upon harvest at different stages. The severity of wilt disease in both susceptible and resistant genotype was observed in longitudinal sections of stem and root vascular region at 15 and 30 DAI (Figure 1). Likewise for SMD, leaf tissue is the specific site of infection and therefore leaf samples of SMD infected genotypes, 'ICP 7035' (SMD resistant) and 'TTB 7' (SMD susceptible) were harvested at 45 and 60 days after sowing (DAS). RNA was extracted and consequently unidirectional cDNA libraries were constructed (see Additional file 1).

### Generation of FW- and SMD- responsive ESTs

**Figure 2:**
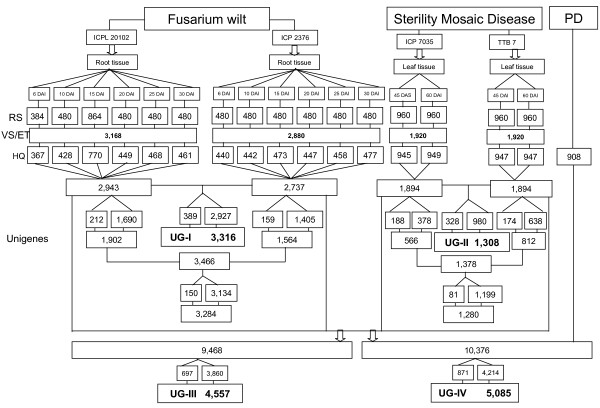
**Summary of total ESTs generated from FW- and SMD- responsive pigeonpea genotypes**. Generation and analysis of ESTs from 16 cDNA libraries of pigeonpea subjected to *Fusarium *wilt (FW) and Sterility mosaic disease (SMD) stresses; (A) Clustering and assembly of 2,943 and 2,737 HQS (High quality sequences) derived from FW-responsive cDNA libraries of pigeonpea genotypes 'ICPL 20102' and 'ICP 2376', respectively resulted in 3,316 unigenes (**UG-I)**; (B) Clustering and assembly of 1,894 HQS from each SMD-responsive pigeonpea genotypes 'ICP 7035' and 'TTB 7' resulted in 1,308 unigenes (**UG-II)**; (C) 9,468 HQS generated from all the four genotypes in the study as shown in (A) and (B) were analyzed together that provided a set of 4,557 unigenes (**UG-III)**; (D) Clustering analysis of generated ESTs in this study along with 908 public domain pigeonpea ESTs, which resulted in 5,085 unigenes (**UG-IV)**, RS: Raw sequences; VS/ET: Vector trimmed/EST trimmed sequences; HQ: High quality sequences; PD: Public domain pigeonpea sequences from NCBI.

A total of 16 unidirectional cDNA libraries were constructed from all the four genotypes i.e. 'ICPL 20102' and 'ICP 2376'; 'ICP 7035' and 'TTB 7' which represent parents of mapping population segregating for FW and SMD, respectively. Using Sanger sequencing approach, 3,168 ESTs were generated from root cDNA libraries of 'ICPL 20102' and 2,880 from 'ICP 2376'. Similarly, 1,920 ESTs were generated from each leaf cDNA libraries of SMD- responsive genotypes, 'ICP 7035' and 'TTB 7'. Details of EST generation from different cDNA libraries are given in Figure 2. In brief, a total of 9,888 ESTs were generated and after stringent screening for shorter (<100 bp) and poorer quality sequences, 9,468 high quality ESTs were obtained, with an average varied-read length of 514 bp (Figure 2). All EST sequences were deposited in the dbEST of GenBank under accession numbers GR463974 to GR473857 and GR958228 to GR958231.

### Pigeonpea EST assembly

**Figure 3:**
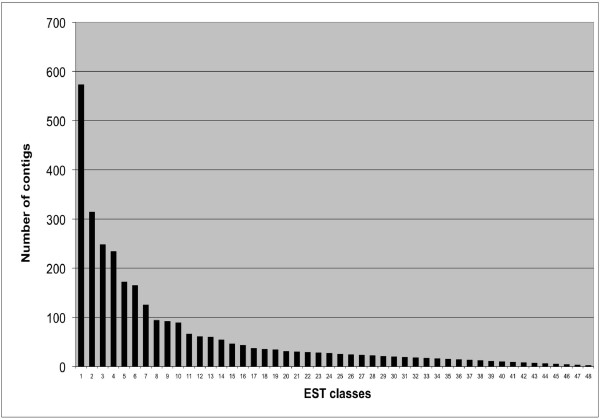
**Frequency and distribution of pigeonpea ESTs among assembled contigs**. Number of ESTs in EST classes (on X-axis) are as following: Class 1=2 , Class 2 = 3, Class 3 = 4, Class 4 = 5, Class 6 = 7, Class 7= 8, Class 8 = 9, Class 9 = 10, Class 10 = 11, Class 11 = 12, Class 12= 13, Class 13 = 14, Class 15 = 16, Class 16 = 17, Class 17 = 18, Class 18 = 19, Class 19 = 20, Class 20 = 21, Class 21 = 22, Class 22 = 23, Class 23 = 24, Class 24 = 25, Class 25 = 27, Class 26 = 28, Class 27 = 29, Class 28 = 30, Class 29 = 31, Class 30 = 34, Class 31 = 35, Class 32= 37, Class 33 = 43, Class 34 = 46, Class 35 = 54, Class 36 = 60, Class 37 = 61, Class 38 = 66, Class 39 = 89, Class 40 = 92, Class 41 = 94, Class 42 = 125, Class 43 = 165, Class 44 = 172, Class 45 = 234, Class 46= 248, Class 47 = 314 and Class 48 = 573.

With an objective to minimize redundancy, clustering and assembly was done for different EST datasets to define unigenes for (a) FW-responsive ESTs, (b) SMD-responsive ESTs, (c) FW- and SMD-responsive ESTs, and (d) the entire set of pigeonpea ESTs including those from the public domain. These unigene (UG) sets were referred to as UG-I, UG-II, UG-III and UG-IV, respectively. The UG-I comprised of 3,316 unigenes with 389 contigs and 2,927 singletons by clustering of 5,680 high quality ESTs. Similarly, for UG-II, clustering of 3,788 high quality sequences resulted in 1,308 unigenes (328 contigs and 980 singletons). Based on clustering of all the 9,468 high quality sequences generated in this study, the UG-III was defined with 4,557 unigenes (697 contigs and 3,860 singletons). The cluster analysis of 908 ESTs available in the public domain along with 9,468 pigeonpea ESTs resulted in UG-IV that included 5,085 unigenes with 871 contigs and 4,214 singletons. The number of ESTs in a contig ranged from 2 to 573, with an average of 7 ESTs per contig. As expected, contigs with two EST members exhibited a higher percentage (46.7%) than contigs with three or more EST members (Figure 3).

### Comparison of pigeonpea unigenes with other plant EST databases

All the four sets of unigenes i.e. UG-I, UG-II, UG-III and UG-IV were analyzed for BLASTN similarity search against available EST datasets of legume species namely chickpea (*Cicer arietinum*), pigeonpea (*Cajanus cajan*), soybean (*Glycine max*), *Medicago *(*Medicago truncatula*), *Lotus *(*Lotus japonicus*), common bean (*Phaseolus vulgaris*) and three model plant species namely *Arabidopsis *(*Arabidopsis thaliana*), rice (*Oryza sativa*) and poplar (*Populus alba*). An E-value significant threshold of ≤ 1E-05 was used for defining a hit. Detailed results of BLASTN analyses for all the four unigenes sets are given in Table 1. For instance, analysis of UG-III found highest identity of 60.3% with soybean, followed by cowpea (43.6%), *Medicago *(43.0%), common bean (42.2%), *Lotus *(37.2%), and the least identity with chickpea (23.2%). Comparative BLASTN analysis of pigeonpea unigenes with EST databases of model plant species showed high identity with poplar (35.4%), followed by *Arabidopsis *(33.7%) and the least similarity with rice (28.3%). Of 4,557 unigenes, 2,839 (62.2%) showed significant identity with ESTs of at least one plant species analysed, while 227 (4.9%) showed significant identity across all the plant EST databases in this study. It is also interesting to note that 39 unigenes did not show any homology with the legume species examined.

**Table 1:** BLASTN analyses of pigeonpea unigenes against legume and model plant ESTs

High quality ESTs generated Unigenes	UG-I 5,680 3,316	UG-II 3,788 1,308	UG-III 9,468 4,557	UG-IV 10,376 5,085
**Legume ESTs**				
Pigeonpea (*Cajanus cajan*) (908)	314 (9.4%)	224 (17.1%)	508 (11.1%)	1,052 (20.6%)
Chickpea (*Cicer arietinum*) (7,097)	585 (17.6%)	507 (38.7%)	1,059 (23.2%)	1,155 (22.7%)
Soybean (*Glycine max*) (880,561)	1,690 (50.9%)	946 (72.3%)	2,750 (60.3%)	2,865 (56.3%)
Cowpea (*Vigna unguiculata*) (183,757)	1,230 (37.0%)	817 (62.4%)	1,988 (43.6%)	2,215 (43.5%)
Medicago (*Medicago truncatula*) (249,625)	1,214 (36.6%)	803 (61.3%)	1,963 (43.0%)	2,153 (42.3%)
Lotus (*Lotus japonicus*) (183,153)	1,015 (30.6%)	738 (56.4%)	1,698 (37.2%)	1,861 (36.5%)
Common bean (*Phaseolus vulgaris*) (83,448)	1,202 (36.2%)	784 (59.9%)	1,927 (42.2%)	2,146 (42.2%)
Significant similarity with ESTs of at least one legume species	1,768 (53.3%)	1,001 (76.5%)	2,757 (60.5%)	3,201 (62.9%)
Significant similarity across legume ESTs	172 (5.1%)	156 (11.9%)	274 (6.0%)	383 (7.5%)
No similarity with legume species	39 (1.1%)	4 (0.3%)	39 (0.8%)	42 (0.8%)
**Model plant ESTs**				
Arabidopsis (*Arabidopsis thaliana*) (1,527,298)	913 (27.5%)	667 (50.9%)	1,536 (33.7%)	1,669 (32.8)
Rice (*Oryza sativa*) (1,240,613)	810 (24.4%)	520 (39.7%)	1,294 (28.3%)	1,389 (27.3%)
Poplar (*Poplus alba*) (418,223)	982 (29.6%)	678 (51.8%)	1,617 (35.4%)	1,753 (34.4%)
Significant similarity with ESTs of at least one Model plant species	1,161 (35.0%)	763 (58.3%)	1,872 (41.0%)	2,019 (39.7%)
Significant similarity across ESTs of all model plant species	635 (19.1%)	460 (35.1%)	1,066 (23.3%)	1,135 (22.3%)
Significant similarity with ESTs of at least one plant species analyzed	1,839 (55.4%)	1,015 (77.5%)	2,839 (62.2%)	3,280 (64.5%)
Significant similarity across ESTs of all plant species analyzed	150 (4.5%)	114 (8.7%)	227 (4.9%)	299 (5.8%)
No similarity with ESTs of any plant species	39 (1.1%)	4 (0.3%)	39 (0.8%)	41 (0.8%)

**Figure 4:**
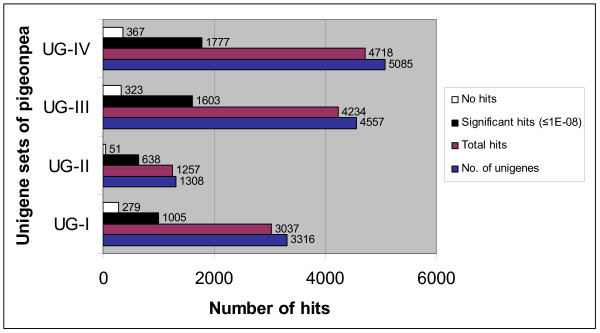
**BLASTX analysis of pigeonpea unigenes against UniProt database**. BLASTX homology search was performed for all the four unigene datasets (UG-I, UG-II, UG-III and UG-IV) against the non-redundant UniProt database. The values against each bar represent total number of unigenes, total number of hits, significant hits at ≤ 1E-08 and no hits for each unigene set.

To identify the putative function of all the unigenes compiled in this study, the unigenes from all the four sets (UG-I, UG-II, UG-III and UG-IV) were compared against the non-redundant UniProt database, using the BLASTX algorithm. At a significant threshold of ≤ 1E-08, 1,005 (30.30%) of UG-I, 638 (48.77%) of UG-II, 1,603 (35.17%) of UG-III and 1,777 (34.94%) of UG-IV unigenes showed significant similarity with known proteins (Figure 4). Details of BLASTX and BLASTN analyses against UniProt database for all four unigene sets are provided in Additional files 2, 3, 4 and 5.

### Functional categorization of pigeonpea unigenes

**Figure 5:**
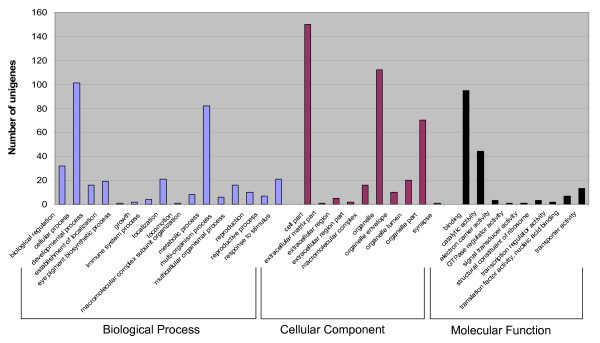
**Gene Ontology (GO) assignment of pigeonpea unigenes (UG-III) by GO annotation**. Functional categorization and distribution of 997 unigenes (UG-III) among three GO categories i.e biological process, cellular component and molecular function according to UniProt database.

The unigenes from all the four sets that showed a significant hit (≤ 1E-08) against the UniProt database were further categorized into functional categories. As a result, 640 (63.6%) of UG-I, 448 (70.2%) of UG-II, 997 (62.1%) of UG-III and 1,119 (62.9%) of UG-IV unigenes were categorised into three principal GO categories i.e. biological process, molecular function and cellular component. Like in earlier studies of this nature, it was observed that one gene could be assigned to more than one principal category, thus the total number of GO mappings from each category exceeded the number of unigenes analyzed. Details on full list of gene annotation for significant hits of four unigene sets are given in Additional file 6, 7, 8 and 9. For instance, of 1,603 (35.1%) unigenes of UG-III, only 997 (21.8%) were assigned to three principle categories. As a result, a total of 132 were grouped under biological process, 132 under molecular function and 153 under cellular component (Figure 5). Under the biological process category, cellular process accounted to 101, followed by metabolic process (82), biological regulation (32) and response to stimulus (21). In the cellular component category, 160 unigenes coded for cell part, 112 to organelle, and 70 to organelle part. In the last category of molecular function, majority of the unigenes were involved in binding (95) and catalytic activity (44). The remaining 606 unigenes which could not be classified into any of the three GO categories were grouped as "unclassified". The distribution of unigenes (UG-III) along with corresponding Gene Ontology (GO) categories are provided in Additional file 10. Based on GO annotation, enzyme commission IDs were also retrieved from the UniProt database to get an overview of unigenes (UG-III) putatively annotated to be enzymes. The major group of unigenes are included under oxidoreductases (107) followed by transferases (91), hydrolases (90), lyases (36), ligases (21) and isomerases (18). Similar patterns of distribution were observed in all the remaining Unigene sets.

### *In silico* expression analysis

**Figure 6:**
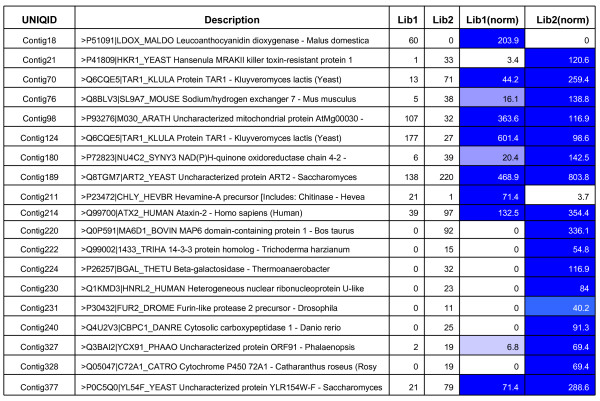
**Differential gene expression between FW- responsive genotypes using IDEG.6 web tool**. Differentially expressed genes between libraries of FW-resistant ('ICPL 20102') and susceptible ('ICP 2376') genotypes. Cells with different degrees of blue color represent extent of gene expression.

**Figure 7:**
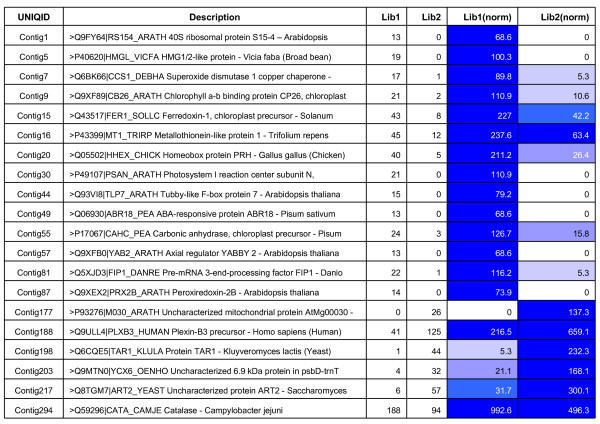
**Differential gene expression between SMD- responsive genotypes using IDEG.6 web tool**. Differentially expressed genes between libraries of SMD resistant ('ICP 7035') and susceptible ('TTB 7') genotypes. Cells with different degrees of blue color represent extent of gene expression.

The identification of differentially expressed genes among specific cDNA libraries of FW- and SMD-responsive genotypes based on EST counts in each contig was done using a web statistical tool IDEG.6. As a result, 19 genes were identified to be differentially expressed between 'ICPL 20102' (FW- resistant) and 'ICP 2376' (FW-susceptible) genotypes, similarly, 20 genes were differentially expressed between 'ICP 7035' (SMD- resistant) and 'TTB 7' (SMD- susceptible) genotypes (Figure 6 and 7).

**Figure 8:**
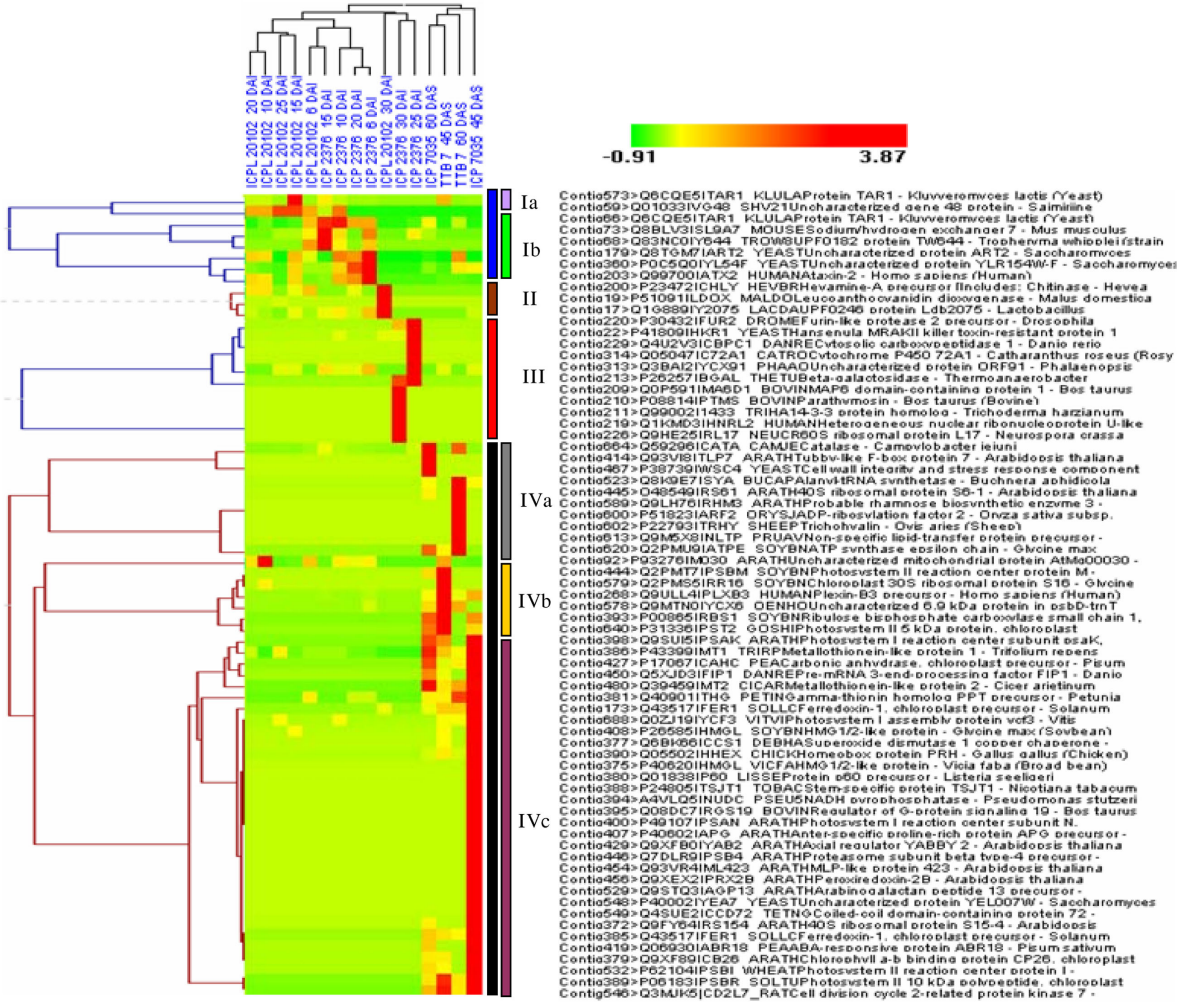
**Hierarchical clustering analysis of differentially expressed genes from 16 libraries of pigeonpea using HCE version 2.0 beta web tool**. Clusters of genes highly expressed in different libraries of pigeonpea genotypes subjected to FW and SMD stress. Columns represent different cDNA libraries and their relationship in a dendrogram. Clustering of highly expressed ESTs (normalized using R statistics, R>8) into four major clusters (indicated in vertical colour bars), and their cluster sub groups based on their library specificity. Colour scale represents the range of expression pattern by different genes with respect to libraries.

To assess the relatedness of each library and expressed genes in terms of expression pattern, a cluster analysis on the basis of EST abundance in each contig was performed [20]. Of the 697 contigs (UG-III), that were subjected to R-statistics [21] only 71 contigs were normalized with a true positive significance (R>8) and were eventually subjected to hierarchical clustering analysis (Additional file 11). The correlated gene expression pattern of all normalized 71 contigs/genes is displayed in Figure 8. All the 12 FW- derived libraries were grouped into a single cluster, while all the four SMD- challenged libraries were grouped into another cluster. About 49 genes were highly expressed in SMD- challenged libraries than in FW- challenged libraries and can be attributed to high accumulation of defence proteins during SMD infection. In the cluster of FW- challenged libraries, the 'ICPL 20102'-30 DAI library was distantly placed between FW- susceptible challenged libraries 'ICP 2376' - 6 DAI and 'ICP 2376' - 30 DAI. Each cluster represents a different pattern of gene expression as shown in Figure 8. Based on the clustering pattern and library specificity, Clusters I and IV were further divided into sub-clusters (represented in different colour bars). The above results indicated that the pattern and percentage of genes expression varied according to severity of the stress in specific library.

In Cluster I, 11.3% (8) of total genes were grouped and further sub divided into two groups with each sharing 2.8% (2) and 8.5% (6) genes, respectively. Similarly, Cluster II and Cluster III accounted for 4.2% (3) and 15.5% (11) genes and the largest Cluster IV, included 69.0% (49) of total genes with three sub groups IVa, IVb and IVc each sharing 14.0% (10), 10% (7) and 45% (32) of genes, respectively. Cluster analysis also showed high level expression of genes related to chloroplast/photosystem related proteins (22.5%), developmental proteins (19.7%), cellular proteins (15.4%), metabolic proteins (14.0%), defence/stimulus responsive proteins (4.3%), protein specific binding proteins (2.8%) and few uncharacterized proteins (19.8%).

### Marker discovery

EST based markers can assay the functional genetic variation compared to other class of genetic markers and hence were targeted for marker development [22]. The unigene set based on generated ESTs in this study as well as the ones available in public domain was used for development of simple sequence repeats (SSR) and single nucleotide polymorphism (SNP) markers.

#### Identification and development of genic microsatellite markers

**Figure 9:**
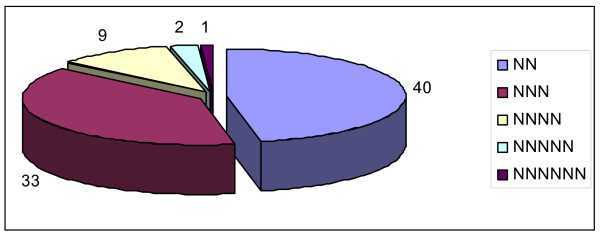
**EST-SSR motifs derived from pigeonpea unigenes (UG-IV)**. Number of EST-SSR repeat motifs (excluding monomers) derived from unigenes (UG-IV) of pigeonpea cDNA libraries subjected to FW and SMD stress.

The entire set of 5,085 pigeonpea unigenes derived from UG-IV was used to identify the SSRs using *MISA *(*MI*cro*SA*tellite) tool [23]. As a result a total of 3,583 SSRs were identified at the frequency of 1/800 bp in coding regions (Table 2). 698 ESTs contained more than one SSR and 1,729 SSRs were found as compound SSRs. In terms of distribution of different classes of SSRs i.e. mono-, di-, tri-, tetra-, penta- and hexa-nucleotide repeats, mononucleotide SSRs contributed to the largest proportion (3,498, 97.6%). Only a limited number of SSRs of other classes were found. For instance, di- and tri- nucleotide SSRs accounted for 40 (1.1%) and 33 (0.9%), respectively. On the other hand, 9 tetrameric, 2 pentameric and 1 hexameric microsatellites were present (Figure 9). While using the criteria for Class I (> 20 nucleotides in length) and Class II SSRs (< 20 nucleotides in length) as used by Temnykh and colleagues [24] and Kantety and colleagues [25], on all SSRs 641 SSRs represented Class I while 2,942 SSRs represented Class II (Table 2).

**Table 2:** Features of SSRs identified in ESTs

SSR database mining			
Total number of sequences examined			5,085
Total length of examined sequences (bp)			2,878,318
Number of ESTs containing SSRs			1,365 (26.8%)
Number of identified SSRs			3,583
Number of sequences containing more than 1 SSR			698
Number of SSRs present in compound formation			1,729
Frequency of SSR			1/0.8 kb
			
Distribution of SSRs			
Type	Class I	Class II	Total
Mono-nucleotides	607	2,891	3,498
Di-nucleotides	10	30	40
Tri-nucleotides	12	21	33
Tetra-nucleotides	9	0	9
Penta-nucleotides	2	0	2
Hexa-nucleotides	1	0	1
Total	641	2,942	3,583

In general, mononucleotide SSRs are not included for primer designing and synthesis. However, as only a very limited number of SSR markers are currently available for pigeonpea in public domain and in a separate study some mononucleotide SSRs were found polymorphic [15], primer pairs were designed for 383 SSRs including mononucleotide SSRs. A total of 94 primer pairs were considered for validation after excluding the primers for monomeric SSR motifs and compound SSRs with mononucleotide repeats. However based on repeat number criteria, such as 5 minimum for di-, tri-, tetra-, penta-nucleotides, primer pairs were synthesized for 84 SSRs. The details of newly developed pigeonpea EST-SSR primers along with corresponding SSR motif, primer sequence, annealing temperature and product size are provided in Additional file 12.

**Figure 10:**
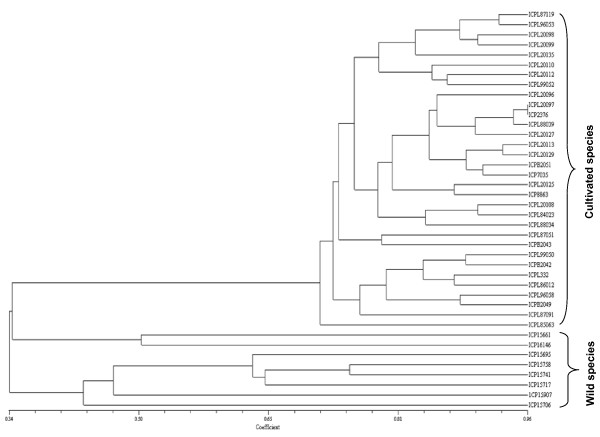
**Dendrogram of elite pigeonpea accessions based on UPGMA analysis**. Unweighted Pair Group Method using arithmetic average dendrogram showing relatedness among the forty elite pigeonpea genotypes representing 8 wild species and 32 cultivated genotypes. The scale at the bottom of the dendrogram indicates the level of similarity between the genotypes.

Newly synthesized 84 markers were analyzed on 40 elite pigeonpea genotypes (Additional file 13). As a result, 52 (61.9%) primer pairs provided scorable amplified products and 26 primer pairs produced a number of faint bands indicative of non-specific amplifications. A total of 15 (28.8%) markers showed polymorphism with 2-7 alleles with an average of 4 alleles per marker in genotypes examined. These markers showed a moderate PIC value ranging from 0.20 to 0.70 with an average of 0.40 (Table 3). To evaluate the genetic variability within a diverse collection of pigeonpea accessions which are parents of different mapping populations segregating for important agronomic traits and also to determine genetic relationship among them, phylogenetic analysis on the basis of dissimilarities was performed using NTSYS software package. The UPGMA cluster diagram showed clear segregation of wild and cultivated species (Figure 10).

**Table 3:** Characteristics of pigeonpea EST-SSR markers

Primer ID	SSR motif	Tm (°C)	Product size (bp)	No. of alleles	PIC value
ICPeM0001	(A)_56_ttg(A)_30_	60	240	1	0.00
ICPeM0003	(A)_23_n(A)_11_n(C)_12_	60	150	7	0.79
ICPeM0005	(A)_99_n(C)_11_	60	280	5	0.35
ICPeM0006	(T)_37_gg(T)_56_	60	246	1	0.00
ICPeM0009	(A)_85_g(A)_27_	60	208	1	0.00
ICPeM0010	(T)_11_n(T)_20_	60	266	1	0.00
ICPeM0011	(A)_58_gggg(A)_24_	60	280	1	0.00
ICPeM0013	(A)_87_g(A)_29_n(C)_11_	62	150	4	0.66
ICPeM0017	(AG)_8_n(AT)_8_	59	240	1	0.00
ICPeM0018	(A)_10_taca(T)_12_	59	90	1	0.00
ICPeM0019	(TTA)_7_n(T)_12_	60	236	1	0.00
ICPeM0023	(A)_128_n(C)_11_n(C)_11_	60	279	1	0.00
ICPeM0024	(A)_11_cccg(A)_10_	60	279	1	0.00
ICPeM0025	(A)_10_n(C)_11_n(C)_11_n(C)_12_	61	223	1	0.00
ICPeM0028	(A)_58_cc(A)_27_	61	239	1	0.00
ICPeM0029	(A)_57_t(A)_30_	60	184	1	0.00
ICPeM0030	(A)_52_tt(A)_28_	61	252	1	0.00
ICPeM0031	(A)_13_gn(A)_67_n(A)_19_	60	279	1	0.00
ICPeM0033	(A)_12_tt(A)_12_n(A)_13_	60	350	7	0.31
ICPeM0034	(A)_13_n(AT)_9_	60	236	1	0.00
ICPeM0035	(T)_21_n(A)_11_	60	218	1	0.00
ICPeM0038	(C)_15_acctcactaaccaaact(C)_10_	^60^	266	1	0.00
ICPeM0039	(G)_10_n(T)_94_	59	262	1	0.00
ICPeM0041	(T)_12_n(A)_10_	60	310	4	0.48
ICPeM0047	(T)_18_c(T)_27_	60	213	1	0.00
ICPeM0050	(A)_112_n(C)_13_n(C)_11_	63	264	1	0.00
ICPeM0052	(C)_12_tccctcctctcgccca(C)_12_	60	233	1	0.00
ICPeM0053	(C)_24_t(C)_26_	60	136	1	0.00
ICPeM0054	(G)_10_agccc(G)_10_	60	<90	1	0.00
ICPeM0060	(T)_13_c(T)_10_	60	150	1	0.00
ICPeM0061	(T)_19_n(A)_28_	59	243	1	0.00
ICPeM0064	(ATT)_7_(T)_10_	59	300	3	0.49
ICPeM0065	(A)_10_(AT)_9_	60	90	1	0.00
ICPeM0066	(AT)_9_	60	310	3	0.34
ICPeM0067	(TA)_11_	60	200	3	0.29
ICPeM0068	(GT)_11_	60	260	4	0.26
ICPeM0069	(AT)_8_	60	<90	1	0.00
ICPeM0070	(AT)_8_	61	310	1	0.00
ICPeM0071	(GA)_9_	61	190	5	0.64
ICPeM0072	(AT)_8_	60	<90	1	0.00
ICPeM0073	(AG)_9_	60	<90	1	0.00
ICPeM0074	(AGA)_6_	60	300	1	0.00
ICPeM0075	(ACA)_6_	60	300	2	0.38
ICPeM0076	(CTT)_6_	60	200	1	0.00
ICPeM0077	(AAT)_7_	60	310	1	0.00
ICPeM0078	(GCC)_6_	60	320	3	0.24
ICPeM0079	(ATT)_6_	60	250	4	0.40
ICPeM0080	(TGGAC)_5_	60	200	1	0.00
ICPeM0081	(TAAT)_5_	60	300	1	0.00
ICPeM0082	(AT)_9_	60	200	3	0.27
ICPeM0083	(AG)_9_	60	190	1	0.00
ICPeM0084	(TATG)_6_	60	240	3	0.59

#### SNP discovery and identification of CAPS markers

**Figure 11:**
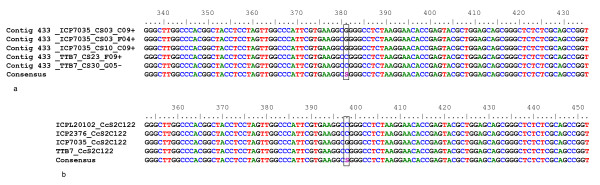
**A snapshot of sequence alignment of EST sequences and amplicons for contig 433 validating the *in silico *predicted SNP**. CAP3 alignment of ESTs (a) generated from 'ICP 7035' and 'TTB 7'in contig 433 showing a SNP between these genotypes and (b) Multiple sequence alignment of amplicon sequences generated from genomic DNA of four genotypes ('ICPL 20102', 'ICP 2376', 'ICP 7035' and 'TTB 7') with the primer pairs for the assembled contig 433. The *in silico *identified SNP in the EST contig 433 was confirmed in the amplicon sequences.

SNPs are an important class of molecular markers which are becoming more popular in recent times. To enhance the reliability of SNPs identification, the SNP which occurred in a contig ≥ 5 ESTs from more than one genotype was considered. *In silico *analysis showed a total of 102 SNPs in 37 (27,659 bp) contigs with a frequency of 1/271 bp (Table 4). With an objective of validating these *in silico *identified SNPs, as an example, 10 contigs were used to generate PCR amplicons and sequence four genotypes namely 'ICPL 20102', 'ICP 2376', 'ICP 7035' and 'TTB 7'. While a scorable and sequenceable amplicon was obtained in case of 6 contigs (contig 210, contig 433, contig 535, contig 555, contig 620 and contig 718), the scorable amplicons were not obtained in case of four contigs (contig 67, contig 330, contig 587 and contig 632). Sequencing of amplicons for all the four genotypes for all the six contigs showed occurrence of SNPs as predicted *in silico *(Additional file 14). For instance, for contig 433, a comparison of the amplified DNA sequences for four genotypes ('ICPL 20102', 'ICP 2376', 'ICP 7035' and 'TTB 7') with the 5 EST sequences coming from two genotypes ('ICP 7035' and 'TTB 7') showed the occurrence of the same SNP G to C between 'ICP 7035' and 'TTB 7' (Figure 11).

**Table 4:** Summary of SNPs and CAPS markers identified from pigeonpea ESTs

Total number of contigs examined (UG-IV)	871
Number of contigs containing ≥ 5 ESTs	133
Number of contigs containing SNPs	37
Total length of 37 contigs (bp)	27,659
Total number of identified SNPs in 37 contigs	102
Average SNP frequency	1/271 bp
Total number of contigs containing CAPS convertible SNPs	37

In order to perform cost-effective and robust genotyping assay for the detected 102 SNPs in 37 contigs, efforts were made to identify the restriction enzymes that can be used to assay SNPs via cleaved amplified polymorphic sequence (CAPS) assay. Results indicated that SNPs present in 37 contigs can be evaluated by using CAPS assay (Table 4).

## Discussion

Plants are known to have developed integrated defence mechanisms against fungal and viral infections by altering spatial and temporal transcriptional changes. The EST approach was successfully utilized in identification of disease-responsive genes from various tissues and growth stages in chickpea [26], *Lathyrus *[27], soybean [28], rice [29] and ginseng [30]. Many earlier studies have shown that resistant genotypes have efficient mechanisms for stress perception and enhanced expression of defence-responsive genes, which maintain cellular survival and recovery [31]. Hence, the present study was undertaken to identify catalog of defence related genes in response to FW and SMD infection in pigeonpea by generating ESTs from different stress challenged tissues at various time intervals.

### Generation of cDNA libraries and unigene assemblies

Roots provide a structural and physiological support for plant interactions with the soil environment by conducting transport of water, ions and nutrients. Plants are encountered with many biotic stress factors which includes bacterial, fungal and viral infection. Roots and leaves are the primary sites of infection by these organisms. Therefore, a total of 16 cDNA libraries were generated at different time intervals to specifically target the roots infected with *Fusarium udum *and leaves infected with SMD. In total 5,680 high quality ESTs were generated from FW- and similarly 3,788 high quality ESTs from SMD- challenged genotypes. Earlier, at the time of analysis in November 2008, the public domain consisted of only 908 ESTs for pigeonpea. Thus the present study contributes approximately 10-fold increase in the pigeonpea EST resource and an addition of 4,557 pigeonpea unigenes (UG-III).

### Functional annotation of pigeonpea unigenes

Homology searches (BLASTN and BLASTX) against other plant ESTs and functional characterization was done for all the defined unigene datasets (UG-I, UG-II, UG-III and UG-IV). Of the 5,085 unigenes (UG-IV) assembled from all the pigeonpea ESTs, 3,280 (64.5%) had significant identity with ESTs of at least one plant species analyzed, 299 (5.8%) unigenes showed significant identity with ESTs of all analyzed plant species in the study, while 41 (0.8%) were found to be novel to pigeonpea. A high significant identity was observed with soybean (56.3%), and the least percentage of similarity was observed with chickpea (22.7%) (Table 1). A similar BLASTN results were observed for the remaining three unigenes sets (UG-I, UG-II and UG-III) against the ESTs of plant species surveyed. Comparative analysis of newly defined UG-III dataset (4,557) with 908 public domain pigeonpea ESTs showed that only 508 (11.1%) shared identity and indicated that our EST sequencing study identified 4,049 (88.9%) new set of pigeonpea unigenes. Relatively, very low similarity of 36.5% with *Lotus *and 42.3% with *Medicago *was observed compared to soybean and cowpea than other legume species. These observations are in accordance with phylogenetic relationships of legumes [32].

The pigeonpea ESTs showed higher similarity to legume ESTs databases (22.7-56.3%) of the legume species than monocot species (27.3-33.4%). Comparative analysis of pigeonpea ESTs with monocot species like rice (27.3%) showed that the percentage of significance is much lower compared to any other legume species, inspite of larger EST repository. This is clearly attributed to phylogenetic divergence between dicots and monocots in course of evolution. These comparisons also indicate that several unigenes that were absent in analysed non-legumes but present in all legume species may be specifically confined to legumes.

BLASTX analyses indicated that those ESTs without significant identity to any other protein sequences in the existing database may be novel and involved in plant defence responses. Hence, this novel EST collection represented a significant addition to the existing pigeonpea EST resources and provides valuable information for further predictions/validation of gene functions in pigeonpea.

A comprehensive comparison of functionally categorized unigenes of all the four unigenes data sets (UG-I, UG-II, UG-III and UG-IV) showed a similar distribution. A large number of unigenes were involved in cell part, organelle, binding, organelle part, metabolic and cellular process among the significantly annotated ones. These observations are consistent with the earlier reported functional categorization studies in rice [29], soybean [33], barley [34] and tall fescue [35]. However, the sequences encoding activities related to categories such as biological regulation and response to stimulus are 28 and 20 incase of FW-responsive ESTs compared to 0 and 2 in case of SMD-responsive ESTs. This was possibly due to the fact that the ESTs generated from FW- challenged root libraries were most abundantly involved in stimulus to pathogenesis and ESTs derived from SMD stress are chloroplast binding proteins. Earlier studies such as Lee and colleagues [36], Ablett and colleagues [37], also reported that photosynthesis-related proteins were the most prevalent from aerial parts of the plant, which would help to make energy related activities such as cell division, growth, elongation and development. Similarly in this study, photosynthesis related genes were identified in larger proportion (30%) in SMD-responsive cDNA libraries derived from leaf tissues.

### *In silico* differential gene expression

The invasion of pathogen not only results in expression of novel genes/transcripts, but also in altering the abundances of different ESTs resulting in induction or repression. This was evident from differential expression of 19 genes between FW-responsive genotypes and 20 genes between SMD-responsive genotypes. It is however, important to mention that *in silico *method of gene expression is not the ideal method to identify the differentially expressed genes. Nevertheless, as large scale EST data were generated from FW- responsive and SMD- responsive genotypes, an effort like some earlier studies [18-21,34] was made to identify some putative genes differentially expressed in FW- and SMD- resistant and sensitive genotypes. Validation of these candidate genes by Northern analysis or real-time quantitative PCR analysis is essential before these candidate genes are deployed in some other studies.

Significant number of unigene sequences related to proteins like kinases, phosphatases, peroxidases, ribonucleases, endochitinases, glucanases and hormones like Abscisic acid responsive (ABR) genes were identified to be differentially expressed and are known to play a vital role in defence mechanism. For example, the cell wall degrading enzymes like endochitinases (EC: 3.2.1.14) implicate a major defence mechanism against pathogen [27]. Similarly, kinases play a major role in the plant's recognition to pathogen [38,39]. For instance, chitinase protein (UniProt ID: P23472), a class of pathogenesis related (PR) proteins with bi-functional role in lysozyme/chitinase activity involved in random hydrolysation of N-aetyl-beta-D-glucosaminide-beta linkages in chitin and chitodextrins during systemic acquired resistance (SAR), was expressed at higher concentrations in FW-responsive resistant genotype ('ICPL 20102') compared to susceptible genotype ('ICP 2376'). The high expression levels of chitinase in resistant genotype indicate the effectiveness within a narrow range of pathogenesis [40,41].

Similarly, the protein coding for ABA-responsive protein (ABR18) (UniProt ID: Q06930), which is involved in stimulus mechanism and cell localization etc. during plant development and one of the vital roles is in defence mechanism during biotic stress signaling. This gene was identified to be expressed relatively higher in SMD-resistant pigeonpea genotype 'ICP 7035' compared to the susceptible genotype 'TTB 7'. During pathogen infection ABA inhibits the transcription of a basic β-1, 3-glucanase (EC: 3.2.1.39) that can degrade the β-1, 3-glucan callose, forming a physical barrier to viral spread through plasmodesmata. This down regulation of β-1, 3-glucanase by ABA can be termed as a resistance factor in plant pathogen interactions [42]. In our study, significant expression signals were observed in SMD resistant genotype 'ICP 7035' during viral infection. This positive correlation between the ABA levels and disease resistance was reported in plant species like common bean [43], rice [44] and tobacco [45]. Different enzymes like methyltransferases (HMT3) (UniProt ID: Q8LAX0) and dehydrogenases (G3PC) (UniProt ID: P34921) are putatively involved in synthesis of lignin in cell walls. These enzymes also play a major role in defence against pathogen interaction [46,47].

An *in silico *hierarchical clustering analysis of 71 differentially expressed and genes across 16 cDNA libraries using HCE V 2.0 was done to infer potential relation between the co-expressed genes. The profiles of some of the interesting gene families and genes that could play an important role in stress stimulus were explained.

In Cluster I, of the 8 contigs, 6 were identified to be highly expressed in FW- challenged libraries of susceptible genotype. The cluster includes genes encoding proteins involved in mitochondrial DNA (mtDNA) stability, Na^+^/H^+ ^exchanger and a few uncharacterized proteins. The sub cluster Ia includes two genes, which are highly expressed in 'ICPL 20102' libraries (15 DAI and 25 DAI). The genes connected in sub cluster Ib are highly expressed in 'ICP 2376' libraries (6 DAI and 15 DAI). One of the putative proteins TAR1 (Transcript Antisense to Ribosomal RNA), a mitochondrial protein is known to be involved in regulation and respiratory metabolism. Over- expression of this protein suppresses the respiration-deficient petite phenotype of a point mutation in mitochondrial RNA polymerase that affects mitochondrial gene expression and mtDNA stability. This dysfunction of mitochondria might occur in response to biotic or abiotic stress [48]. The over-expression of these genes was observed only in 15 DAI libraries of both the FW- responsive genotypes. And their immediate disappearance in the later stages of infection in resistant genotype libraries and continued expression in susceptible genotypes supports a hypothesis that continual expression of this protein may lead to mitochondrial dysfunction and subsequent cell degeneracy.

Another protein Na^+^/H^+ ^exchanger 7 (UniProt ID: Q8BLV3) is an ubiquitous ion transporter that serves multiple cell physiological processes such as intracellular pH homeostasis and electro neutral exchange of protons for Na^+ ^and K^+ ^across endomembranes. Biochemical studies suggest that Na^+^/H^+ ^exchangers in the plasma membrane of plant cells contribute to cellular sodium homeostasis during salt stress, although the above protein is expressed in high salt stressed plants, it may also be expressed during biotic stress. Its high expression in susceptible genotype 'ICPL 2376' at 6 and 15 DAI libraries shows the severity of stress during fungal pathogenesis. And in the remaining FW- and SMD- challenged libraries this shows normal expression.

Cluster II genes include hevamine (EC: 3.2.1.14) and Leucoanthocyanidin dioxygenase (EC: 1.14.11.19) specifically expressed in 'ICPL 20102' 30 DAI library. The important protein hevamine represents a new class of polysaccharide-hydrolyzing (βα)_8 _barrel enzyme belonging to families of plant chitinases and lysozymes, which are vital for plant defence against pathogenic bacteria and fungi. Recent results indicate that these enzymes may be involved not only in defence-related process or general stress response but also in growth and development processes [49]. The high expression of these proteins in the late phase of *Fusarium *infection indicates their prolonged defensive role against fungal pathogenesis.

The genes connected in Cluster III are highly expressed in FW-challenged 'ICP 2376' - 25 and 30 DAI libraries. The genes related to endoprotease activity, beta-glucan synthesis, carboxypeptidases, alkaloid biosynthesis, secologanin biosynthesis, beta-galactosidase activity, microtubule-stabilizing activity, nucleic acid binding protein, ribonucleoprotein and a few uncharacterized proteins were constituted in this cluster. For instance, antifungal class proteins such as beta-glucanases (EC: 3.2.1.39) and *Hansenula mrakii *killer toxin-resistant proteins (UniProt ID: P41809) located in epidermal leaf cells are believed to be involved in cell differentiation and defence against fungal pathogens [50]. Plants deficient in these enzymes generated by antisense transformation showed markedly reduced resistance to viral and fungal infection. Similarly, another class of proteins carboxypeptidases (EC: 3.4.16 - 3.4.18) with diverse functions ranging from catabolism to regulating biological processes, including function as a defence against pathogen attack [51].

The majority of genes (49) segregated in Cluster IV were highly expressed in SMD-responsive cDNA libraries derived from leaf tissues. In total Cluster IV genes showed high gene expression in SMD derived libraries. As expected, photosynthesis related transcripts were abundantly represented in sub clusters IVa, IVb and IVc, and these include putative transcripts like ribosomal proteins, mitochondrial proteins, chloroplast precursor proteins, photosystem I and II reaction centre proteins. The observed expression pattern of photosynthesis related proteins in this study is also consistent with experimental observations in barley [34].

Overall, the differentially expressed genes are involved in diverse pathways, displaying complex expression patterns. The different clusters based on monitoring of gene expression patterns propose that various pathways in response to biotic stress exist in pigeonpea and their interaction can lead to differential stress tolerance. The uncharacterized class of transcripts co-expressed could be repository of novel proteins and further characterization of these may reveal their significant role in plant stress responses [52].

### Development of functional markers

One of our primary goals of our research programme is to develop molecular markers based on expressed sequences and screen them for polymorphism. During the last decade, microsatellites or SSRs have proven to be useful markers in plant genetic research and have been used for marker-assisted breeding purposes. The presence of SSRs in the coding region suggests their importance as functional or gene based markers [1,11,53]. Unfortunately, development of microsatellite markers is expensive, labor intensive, and time consuming if they are being developed from genomic libraries [54]. The data mining of microsatellites markers from EST data can be a cost effective option. The cost of mining EST libraries is far lower than other traditional methods, and SSR development from ESTs has been successful in EST data mining [22,23,53-56]. SSR motifs with repeats more than eight for di-nucleotides, six for tri-nucleotides, and five for tetra-nucleotides were considered. Dimeric repeat motifs (40) were relatively abundant than trimeric repeats (33). In addition to this, tetra-, penta- and hexameric repeat motifs were considerably less represented. A total of 94 SSR markers have been synthesized and characterized for polymorphism survey. However, there are some distant contrasts in frequency and distribution of SSRs in ESTs and in genomic survey sequences (GSSs). In general di-nucleotide SSRs of all repeat lengths are more common in GSSs and tri-nucleotide SSRs are common in the ESTs [22,23,56,57]. As against these reports, in our findings we observed that di-nucleotide repeats are more abundant than tri-nucleotide repeat motifs [58,59]. However this observation is not unexpected as the frequency and distribution of SSR depends on several factors such as size of dataset, tools and criteria used for SSR discovery [22].

In this study, a total of 15 polymorphic EST-SSRs primer pairs were validated and used for diversity study on forty pigeonpea genotypes representing 32 cultivated (*C. cajan*) and 8 wild species (six *C. scarabaeoides *and two *C. platycarpus*). All markers detected at least one allele in all genotypes tested, suggesting transferability of all markers across the *Cajanus *genus. In addition to high transferability, EST-SSRs are good candidates for the development of conserved orthologous sequence (COS) markers for genetic analysis and breeding of different species [10]. However, EST-SSRs were reported to be less polymorphic than genomic SSRs in crop plants due to greater DNA sequence conservation in transcribed regions [22,60]. For instance, the 15 SSR loci provided only 60 alleles with an average of 4 alleles per loci and an average 0.43 PIC value. Similar kind of diversity features were observed in earlier SSR based diversity studies in pigeonpea [14,15].

EST-SSR profiles obtained on 40 pigeonpea genotypes were used to compute pair-wise genetic distances among different genotypes to construct a dendrogram based on UPGMA clustering. The neighbor joining tree grouped 40 pigeonpea genotypes into three major clusters (Figure 10). The Cluster I comprising 32 genotypes (cultivated) is the largest cluster followed by Cluster III containing six wild genotypes (*C. scarabaeoides*) and Cluster II is the smallest cluster with two wild genotypes belonging to *C. platycarpus *species revealing clear segregation of the cultivated and the wild species. Less genetic variation was detected with in cultivated species, with only nine markers detecting polymorphism and a total of 35 alleles. The low genetic variability amongst cultivars when compared with the wild species genotypes suggests that natural and artificial selection has contributed to the selection of specific alleles and to changes of allelic frequencies at specific loci as reported by Odeny and colleagues [14]. The distinctness of *C. platycarpus *with *C. scarabaeoides *accessions observed in this study correlate well with earlier studies [61]. It is also important to note that 'ICPL 20097' and 'ICP 2376' genotypes were found closely related with high genetic similarity as both of these genotypes belong to the same geographic region. In conclusion, EST-SSR markers developed in this study complement the currently available or ongoing efforts on development of genomic SSRs that will be a valuable resource for linkage map development and marker assisted selection in pigeonpea [12].

SNPs and indels are an essentially inexhaustible resource of polymorphic markers for use in the high-resolution genetic map development of traits and for association studies. Although a variety of molecular markers are available SNPs are comparatively advantageous because of their abundance and amenability to high throughput approaches [62]. In addition, SNPs also offer several advantages like high-throughput and cost-effective genotyping [63] and identification of functional/gene-based markers for complex trait through linkage map development or association genetics [9,10,64,65]. Although SNP discovery was a cost effective task in past, advances in next generation sequencing technologies have made SNP discovery cheaper and faster [66]. However in case for a given species, ESTs are available from more than one genotype, *in silico *mining of ESTs is still a very inexpensive and fast approach for SNP discovery [17,64] and therefore we used this approach for mining SNPs in this study.

By using *in silico *mining approach in a total of 871 contigs coming from 10,376 ESTs (9,888 generated in this study and 908 available in public domain), a total of 102 potential SNPs were identified in 37 contigs that were consisted of ≥ 5 ESTs. Smaller contigs were not considered for SNP mining as these contigs are prone to errors due to lack of read depth as reported by Wang and colleagues [67]. Sequence analysis of PCR products for a subset of 6 out of 10 contigs confirmed the occurrence of SNPs in all the cases. As PCR products could not be generated for remaining four contigs, the presence of SNPs could not be confirmed in those cases. Furthermore, as SNP genotyping is another important criteria in breeding programmes, identification of CAPS markers for 37 contigs will facilitate SNP genotyping even in low tech laboratories [63].

## Conclusion

This study has contributed a new and significant set of 9,888 ESTs that together with 908 public domain ESTs provides a unigene set of 5,085 sequences for pigeonpea. Detailed analysis of these datasets have provided several important features of pigeonpea transcriptome such as conserved genes (across legumes and model plant species) as well as possible pigeonpea specific genes, assignment of pigeonpea genes to different GO categories, identification of differentially expressed genes in response to FW- and SMD- stresses, etc. In terms of applied aspect of developed resource in breeding, this study has demonstrated development and application of gene-based molecular markers i.e SSRs, SNPs and CAPS. In summary, it is anticipated that this study is a significant contribution to enhance genomic resources in a so called orphan legume crop that will eventually impact pigeonpea breeding [11,12].

## Methods

### Plant material

Four pigeonpea genotypes namely 'ICPL 20102' (FW- resistant), 'ICP 2376' (FW- susceptible), 'ICP 7035' (resistant to SMD) and 'TTB 7' (highly susceptible to SMD) were used for constructing the cDNA libraries and generating the ESTs. Seeds of two genotypes ('ICPL 20102', 'ICP 2376') were procured from Legume Pathology section at ICRISAT and for the remaining two genotypes ('ICP 7035' and 'TTB 7') were obtained from Dr. M Byregowda, University of Agricultural Sciences, Bangalore, India.

A total of 40 genotypes including 32 genotypes from cultivated species (*C. cajan*) and 8 genotypes from 2 wild species (*C. platycarpus *and *C. scarabaeoides*) were used for validation and diversity analysis with new set of EST-SSR markers. These genotypes were obtained from Pigeonpea Breeding (Dr. KB Saxena) and Genebank (Dr. HD Upadhyaya) and have been listed in Additional file 13.

### Inoculation treatment for FW and SMD

Seeds of FW-tolerant ('ICPL 20102') and FW-susceptible ('ICP 2376') were germinated in 15-inch deep polythene covers filled with sterile soil and sand (1:1) in a glass house at 23 ± 3°C under 80% relative humidity. The root, being the primary target of the pathogen *Fusarium udum *and the possible site of the initial defence response, was selected as the tissue of study. Ten days old seedlings were uprooted from pots and the root system was thoroughly washed in running tap water and rinsed with distilled water. Seedlings of each genotype were inoculated by immersing the roots for 2 min in fungal inoculum (*Fusarium udum *culture). The spore suspension at 6 × 10^5 ^conidia/ml, was made by adding fungal spores from several culture plates (*Fusarium *was grown on potato dextrose media supplemented with 0.25 μg/ml tetracycline). Immediately following the inoculation, the seedlings were transplanted to sterilized sand and soil mixture (1:1) in pots and were transferred to glass house.

In order to capture the genes expressed in resistant and susceptible genotypes at different time periods after inoculation, six stages i.e. 6, 10, 15, 20, 25 and 30 days after inoculation (DAI) were selected arbitrarily to construct the cDNA libraries. From 6-15 DAI, chlorosis symptoms were observed on the leaves and aerial parts of plant material, indicated the severity of *Fusarium *wilt disease. Furthermore, from each stage of days after inoculation, shoot and root section cuttings were made and observed the fungal penetration into the vascular tissues. Based on microscopic observations at different stages, initial symptoms of fungal infection were noticed in root vascular tissue at 15 and 20 DAI stages. Beyond 20 DAI, though the fungus penetrates deeper into the vascular tissues of susceptible and resistant varieties to some extent, the susceptible variety shows complete *Fusarium *symptoms where as the resistant variety prevents most of the attacking fungus from reaching maturity and developing symptoms.

For SMD study, highly susceptible ('TTB 7') and resistant ('ICP 7035') pigeonpea genotypes that are parents of a mapping population segregating for resistance to SMD were chosen. Forty seeds from each accession were sown in plastic bags filled with sterilized soil and were maintained in a glass house under optimal physiological conditions as described above. Ten days after sowing, the aerial parts of the seedlings were stapled with mosaic virus infected leaves. The viral disease is caused by pigeonpea sterility mosaic virus (PPSMV) and transmitted by an eriophyid mite *Aceria cajani *Channabasavanna [7]. The disease slowly spreads into the vascular tissues from the aerial parts through mite population which is characterized by a bushy and pale green appearance of plants. Based on the severity of disease symptoms, leaves with visible SMD lesions were harvested at 45 and 60 days after sowing (DAS) stages for construction of cDNA libraries.

### cDNA library construction

Root and leaf tissue samples were collected from FW- and SMD- responsive genotypes at different time-points till the infection stage reached stagnant phase. RNA was isolated from the above two tissue samples according to the protocol described by Schmitt and colleagues [68]. RNA quality was assessed using formamide gel electrophoresis and poly (A)^+ ^RNA was isolated with poly (A) tract mRNA isolation system IV (Promega, Madison, WI, USA) as described by the manufacturers. Double-strand cDNA was constructed using Super SMART™ PCR cDNA Synthesis kit (Clontech^®^, Mountain View, CA, USA) as described in the manufacturer's instructions. The resulting cDNA was size fractioned on 1.2% agarose gel. cDNA fractions containing fragments greater than 500 bp were selected for library construction. Subsequently, the cDNA was ligated into pGEM^® ^Easy vector (Promega^®^, Madison, WI, USA) and ligation was allowed to proceed overnight at 14°C. The resulting plasmids were electroporated using One Shot^® ^Top 10 Electrocomp™ cells (Invitrogen, Carlsbad, CA, USA). The transformants were spread on LB Agar plates containing 100 mg/ml ampicillin for direct picking. Based on blue/white screening, recombinant clones were picked into Nunc-Immuno™ 96 MicroWell™ Plates (Nunc™, Roskilde, Denmark) containing LB broth with 100 μg/ml ampicillin and grown for overnight at 37°C on a rotary shaker at 220 rpm. Glycerol stocks in 96-well format were prepared by combining 38 μl of 60% (v/v) glycerol with 150 μl of culture and frozen at -80°C.

### EST sequencing, editing and assembly

Clones were randomly selected and on an average of 500 clones were sequenced per library in case of FW-response study and 1000 clones per library in case of SMD-responsive study. The plasmid DNA from these clones (i.e. colonies) was extracted using a 96-well alkaline lysis method prior to sequencing [69]. Plasmid DNA sequencing was performed by commercial DNA sequencing service provider (Macrogen Inc., Korea) using the standard M13 forward primer.

The FASTA files containing the raw sequences were edited by the software Sequencher™ 4.0 (Gene Codes Corporation, Ann Arbor. MI, USA) to remove the vector sequences. The vector screened sequences were subjected to EST trimmer [70], to trim poly-A ends and low quality sequences. High quality sequences of >100 bp were selected for further sequence analysis. ESTs were clustered and aligned into contigs and singletons using the CAP3 program [71].

In order to assess the number of unique and overlapping transcripts among the 16 libraries, four data sets were generated; those derived from libraries constructed from of FW-responsive genotypes (UG-I); those derived from libraries constructed from of SMD-responsive genotypes (UG-II); combined dataset of FW- and SMD- responsive ESTs (UG-III); and also from public domain sequences with total generated ESTs in this study (UG-IV). In addition to the above assembly of unigene sets, CAP3 analysis was also performed to libraries derived from FW- resistant genotype, FW- susceptible genotype, SMD- resistant genotype and from SMD- susceptible genotype individually.

### Homology search and functional annotation

The unigene sequences were also characterized for nucleotide homology search against the EST datasets of selected legume species [pigeonpea (*Cajanus cajan*)-908, chickpea (*Cicer arietinum*)-7,097, soybean (*Glycine max*)-880,561, *Medicago *(*Medicago truncatula*)-249,625, common bean (*Phaseolus vulgaris*)-83,448, cowpea (*Vigna unguiculata*)-183,757 and *Lotus *(*Lotus japonicus*)-183,153] and selected model plant species [rice (*Oryza sativa*)-1,240,613, *Arabidopsis *(*Arabidopsis thaliana*)-1,527,298 and poplar *(Populus alba*)-418,223] available at National Center for Biotechnology Information (NCBI, http://www.ncbi.nlm.nih.gov) using BLASTN algorithm [72]. A match was considered significant at E-value ≤ 1E-05.

Each unigene dataset was subjected to BLASTX analysis against the non-redundant protein database of UniProt to deduce a putative function. Sequence similarity was considered as significant at E-value ≤ 1E-08. Each unigene was assigned a putative cellular function based on the significant database hit with lowest e-value. Subsequently, unigenes that showed a significant BLASTX hit were used for functional annotation based on Gene Ontology categories from UniProt database (UniProt-GO). This process allowed assignment of unigenes to the GO functional categories of biological process, cellular component and molecular function. Distribution of unigenes was further investigated in terms of their assignment to sub-categories of the main GO categories.

### In silico expression and hierarchical clustering

In order to identify the differentially expressed genes in FW- and SMD- responsive genotypes, 389 contigs coming from FW-responsive genotypes and 328 contigs coming SMD-responsive genotypes were analyzed by using IDEG.6 web interface tool [73,74]. The IDEG.6 web tool allows running six different statistical analyses for the detection of differentially expressed genes in multiple tag experiments. For pair-wise comparisons, the Audic and Claverie test, Fisher exact test and chi-square tests (Χ^2^) were used and in multiple comparisons R- statistics test, Greller and Tobin test and chi-square tests (Χ^2^) were used [73,74].

Further, gene expression analysis was performed with hierarchical clustering expression (HCE) version 2.0 beta software [75] using transcript abundance data from UG-III set that includes 697 contigs derived from both the stress responsive libraries. As a pre-requisite for HCE analysis, all 697 contigs were subjected to R statistics (R>8) and only those contigs (71) were selected that have; (i) minimum 5 ESTs, and (ii) differential abundance of ESTs coming from different libraries. The matrix file developed based on the frequency of ESTs to each of 71 contigs was used as input file for above mentioned HCE tool.

### Identification and development of SSR markers

A total of 5,085 unigenes (unigene set, UG-IV) developed based on 9,888 ESTs generated in this study and 908 public domain ESTs were searched with a Perl script program, *MISA *(*MI*cro*SA*tellite) [23,76] for identification and localization of SSRs. The SSR motifs, with repeat units more than five times in di-, tri-, tetra-, penta- and hexa- nucleotides were considered as SSR search criteria in *MISA *script. The Primer3 programme [77] was used for designing the primer pairs for SSRs and custom synthesized by MWG (MWG-Biotech AG, Bangalore, India).

The primer pairs for SSRs were tested for their utility as potential genetic markers on 40 elite genotypes of pigeonpea (Additional file 13). PCR amplifications were performed in 5 μl reactions containing 5 ng of genomic DNA, 1× SE-Taq DNA polymerase buffer (including 1.5 mM MgCl_2_), 2 mM dNTPs, 10 pmol of each primer and 0.1 U *Taq *DNA polymerase (*SibEnzyme*, Novosibirsk, Russia) with the following touch down profile; 3 min at 95°C; 5 cycles of 20 sec at 94°C, 20 sec at 60°C minus 1°C/cycle, 30 sec at 72°C; 40 cycles of 20 sec at 94°C, 20 sec at 56°C, 30 sec at 72°C; and 20 min at 72°C for final extension. PCR products were separated on 6% non-denaturing polyacrylamide gels for 3 h at 600 V and visualized by silver staining. The polymorphism information content (PIC) of individual EST-SSR markers was calculated by using the standard formula [62]. Only data from polymorphic SSR loci were used for diversity analysis. Genetic similarities between any two genotypes were estimated according to Nei and Li [78]. All 40 genotypes were clustered with the Unweighted Pair Group Method using arithmetic average (UPGMA) in the SAHN procedure of the NTSYS-PC v2.10t [79].

### SNP detection and their conversion into CAPS

All 871 contigs obtained from the collection of 5,085 unigenes (UG-IV) were searched for putative SNP/indels by using an integrated pipeline for large scale SNP discovery [80,81]. The pipeline utilized the CAP3 output files as input to detect SNPs/indels based on the nucleotide redundancy in the multiple sequence alignments. The auto SNP pipeline generated text file includes contig ID, number of sequences in the contig ID, consensus length, number of SNPs, mutation type and SNP frequency. The threshold for identification of SNPs was based on the number of sequences (≥ 5) in each consensus sequence and two or more sequences from different genotype. In order to verify the SNPs at sequence level, the PCR amplicons of all four genotypes were sequenced using the corresponding forward and reverse primers for a set of 10 contigs (see Additional file 14). The amplicons were purified and further sequencing was done as described [80]. The sequenced data along with the sequences of ESTs (that provided the SNPs initially) were aligned and analyzed using BioEdit programme http://www.mbio.ncsu.edu/BioEdit/bioedit.html.

For converting SNPs into cleaved amplified polymorphic sequence (CAPS) markers, SNPs present in 37 contigs were analyzed to identify the recognition site for any of commercially available 725 restriction enzymes [82] by using integrated SNP2CAPS pipeline [80].

## Authors' contributions

NLR, BNG and PL conducted experiments, NLR, BJ, PJH and RKV analyzed EST data, SP and MB contributed plant tissues to generate ESTs, BJ, NKS and RKV provided scientific inputs to analyze and interpret results, NLR, PJH and RKV wrote the manuscript in consultation with other co-authors, RKV conceived, planned coordinated and supervised the overall study and finalized the manuscript. All authors read and approved the final manuscript.

## Supplementary Material

Additional file 1 **Sterility mosaic disease (SMD) responsive pigeonpea seedlings**. a) Sterility mosaic disease infected pigeonpea genotypes 'ICP 7035' and 'TTB 7' at 45 days after sowing (DAS); initiation of SMD infection to the aerial parts of susceptible genotype 'TTB 7'; b) Severe SMD infection observed in the susceptible genotype ('TTB 7') showing pale green and bushy aerial parts after 60 DAS as against resistant genotype (ICP 7035). Click here for file

Additional file 2 **BLASTX and BLASTN result of UG-I dataset**. Table provides BLASTX and BLASTN results of UG-I dataset. Click here for file

Additional file 3 **BLASTX and BLASTN result of UG-II dataset**. Table provides BLASTX and BLASTN results of UG-II dataset. Click here for file

Additional file 4 **BLASTX and BLASTN result of UG-III dataset**. Table provides BLASTX and BLASTN results of UG-III dataset. Click here for file

Additional file 5 **BLASTX and BLASTN result of UG-IV dataset**. Table provides BLASTX and BLASTN results of UG-IV dataset. Click here for file

Additional file 6 **Gene Ontology categorization for UG-I dataset**. Table shows classification of significant BLASTX hits (≤ 1-08) of UG-I dataset to different Gene Ontology categories. Click here for file

Additional file 7 **Gene Ontology categorization for UG-II dataset**. TTable shows classification of significant BLASTX hits (≤ 1-08) of UG-II dataset to different Gene Ontology categories. Click here for file

Additional file 8 **Gene Ontology categorization for UG-III dataset**. Table shows classification of significant BLASTX hits (≤ 1-08) of UG-III dataset to different Gene Ontology categories. Click here for file

Additional file 9 **Gene Ontology categorization for UG-IV dataset**. Table shows classification of significant BLASTX hits (≤ 1-08) of UG-IV dataset to different Gene Ontology categories. Click here for file

Additional file 10 **Gene Ontology categorization for UG-III dataset**. Tables showing significant hits (≤ 1E-08) of unigenes from four pigeonpea unigene dataset (UG-III) and its corresponding Gene Ontology categories: a) Biological process b) Cellular component c) Molecular function. Click here for file

Additional file 11 **Hierarchical clustering of UG-III contigs**. Table showing data matrix of 71 contigs as four clusters, represented in Hierarchical clustering dendrogram with corresponding number of ESTs represented from each library. Click here for file

Additional file 12 **List of newly developed pigeonpea EST-SSRs**. List of newly developed pigeonpea EST-SSR markers with corresponding details of primer ID, SSR motif, primer sequence, melting temperature and product size. Click here for file

Additional file 13 **List of pigeonpea elite genotypes used for diversity assessment**. List of pigeonpea accessions used in assessment of newly synthesized EST-SSR markers with corresponding details of species name, geographical origin, type. Click here for file

Additional file 14 **Validation of *in silico *identified SNPs in EST contigs through sequencing**. Validation experiments of *in silico *identified SNPs have been shown in this file for 10 contigs. Click here for file
