# Supplementary figures and images for: The first set of EST resource for gene discovery and marker development in pigeonpea (Cajanus cajan L.)
Source: BMC Plant Biol. 2010 Mar 11;10:45. doi: 10.1186/1471-2229-10-45 (PMC2923520; doi:10.1186/1471-2229-10-45)

## Slide 1
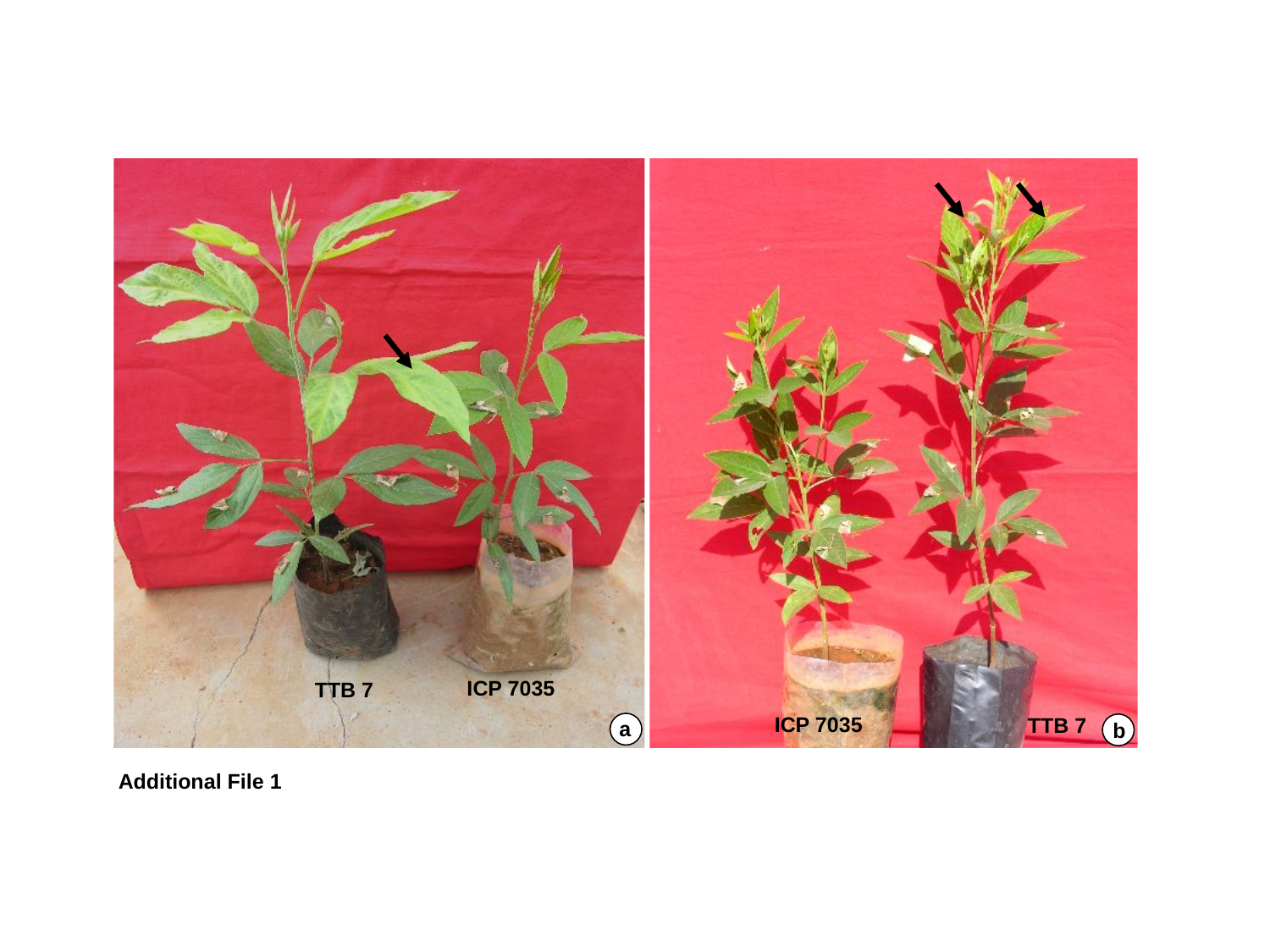

ICP 7035
TTB 7
ICP 7035
TTB 7
a
b
Additional File 1

Supplement: Additional file 1 — Sterility mosaic disease (SMD) responsive pigeonpea seedlings. a) Sterility mosaic disease infected pigeonpea genotypes 'ICP 7035' and 'TTB 7' at 45 days after sowing (DAS); initiation of SMD infection to the aerial parts of susceptible genotype 'TTB 7'; b) Severe SMD infection observed in the susceptible genotype ('TTB 7') showing pale green and bushy aerial parts after 60 DAS as against resistant genotype (ICP 7035). [file 1471-2229-10-45-S1.PPT]

## Slide 1
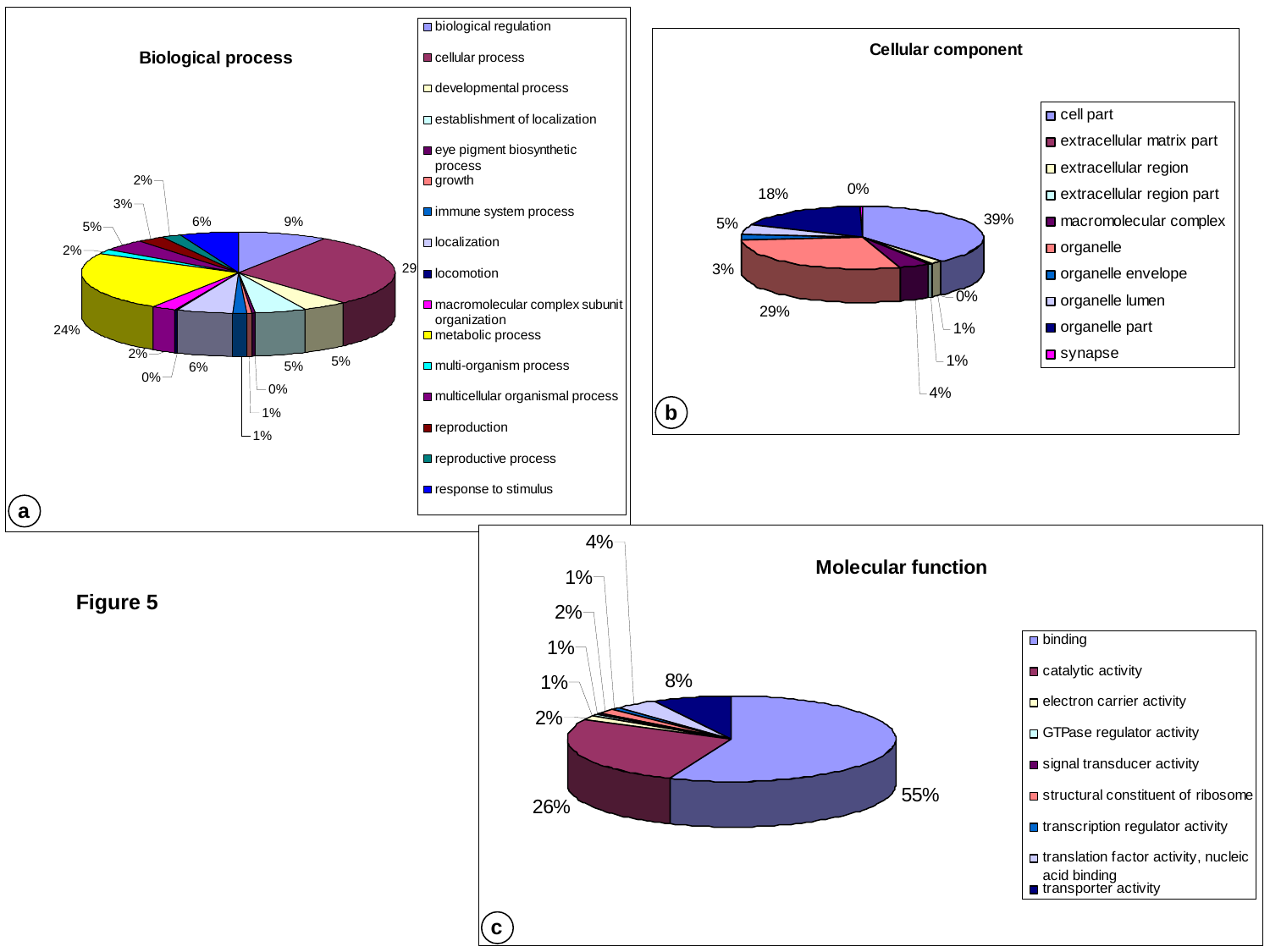

b
a
Figure 5
c

Supplement: Additional file 10 — Gene Ontology categorization for UG-III dataset. Tables showing significant hits (≤ 1E-08) of unigenes from four pigeonpea unigene dataset (UG-III) and its corresponding Gene Ontology categories: a) Biological process b) Cellular component c) Molecular function. [file 1471-2229-10-45-S10.PPT]
